# The Role of N-Acetylcysteine in Obsessive-Compulsive (OCD) and Related Disorders

**DOI:** 10.1192/j.eurpsy.2022.1653

**Published:** 2022-09-01

**Authors:** J. Sá Couto, J. Rodrigues, M. Pão Trigo, B. Da Luz, T. Ventura Gil

**Affiliations:** Centro Hospitalar Universitário do Algarve, Departamento De Psiquiatria E Saúde Mental, Unidade De Faro, Faro, Portugal

**Keywords:** Compulsion, obsessive-compulsive disorder, Glutamate, N-acetylcysteine

## Abstract

**Introduction:**

N-acetylcysteine is known for its uses in non-psychiatric conditions, such as paracetamol overdose and as a mucolytic. The rationale for its administration in psychiatric conditions is based on its ability reducing synaptic glutamate release, which was found to be increased in the cerebrospinal fluid of OCD patients.

**Objectives:**

Evaluating N-acetylcysteine efficacy in OCD symptoms. Studying mechanisms underlying its action. Identifying the frequency of side effects.

**Methods:**

*PubMed* database search, with the *“N-acetylcysteine obsessive compulsive”* keyword expression. The search was restricted to English-only articles, published in the last ten years. Twenty-five results among the best match correspondence were selected. Reference lists of articles were reviewed to identify additional articles.

**Results:**

Oliver *et al.* found that a daily dose of 2.400 to 3.000 milligrams of N-acetylcysteine reduced the severity of obsessive-compulsive symptoms with minimal side effects; Smith *et al.* found inconclusive evidence on its efficacy. A clinical trial from Ghazinadeh *et al.* revealed N-acetylcysteine to be effective as an add-on to citalopram, reducing the score of resistance/control to obsessions after supplementing with N-acetylcysteine. Costa *et al.* found out it was superior to placebo in anxiety control as a secondary outcome.

**Conclusions:**

The potential efficacy of N-acetylcysteine in the treatment of psychiatric disorders attracted interest. Mixed evidence was found that N-acetylcysteine may have some benefits controlling compulsions, both as an adjunctive as and as monotherapy. Thus, larger and more robust studies are required to further investigate the clinical effectiveness of N-acetylcysteine in this area.

**Disclosure:**

No significant relationships.

